# 5,5-Dimethyl-2,2-bis­(pyridin-2-yl)-1,3-diazinane

**DOI:** 10.1107/S1600536812021629

**Published:** 2012-05-19

**Authors:** Ismail Warad, Afaf Alruwaili, Saud I. Al-Resayes, M. Iqbal Choudhary, Sammer Yousuf

**Affiliations:** a Department of Chemistry, College of Science, King Saud University, PO Box 2455, Riyadh 11451, Saudi Arabia; b Department of Chemistry, College of Science, University of Hail, PO Box 2440, Hail, Saudi Arabia; c H.E.J. Research Institute of Chemistry, International Center for Chemical and Biological Sciences, University of Karachi, Karachi 75270, Pakistan

## Abstract

In the mol­ecule of the title compound, C_16_H_20_N_4_, the 1,3-diazinane ring adopts a chair conformation and the dihedral angle formed by the pyridine rings is 78.64 (8)°. The mol­ecular conformation is stabilized by an intra­molecular C—H⋯N hydrogen bond, forming an *S*(6) ring motif. In the crystal, centrosymmetrically related mol­ecules are linked into dimers by pairs of N—H⋯N hydrogen bonds, generating rings of *R*_2_^2^(10) graph-set motif.

## Related literature

For the structures of related hexa­hydro­pyrimidines, see: Al-Resayes (2009 ▶); Song *et al.* (2010 ▶); Jayaratna & Norman (2010 ▶); Fun & Kia (2008 ▶). For standard bond lengths, see: Allen *et al.* (1987 ▶).
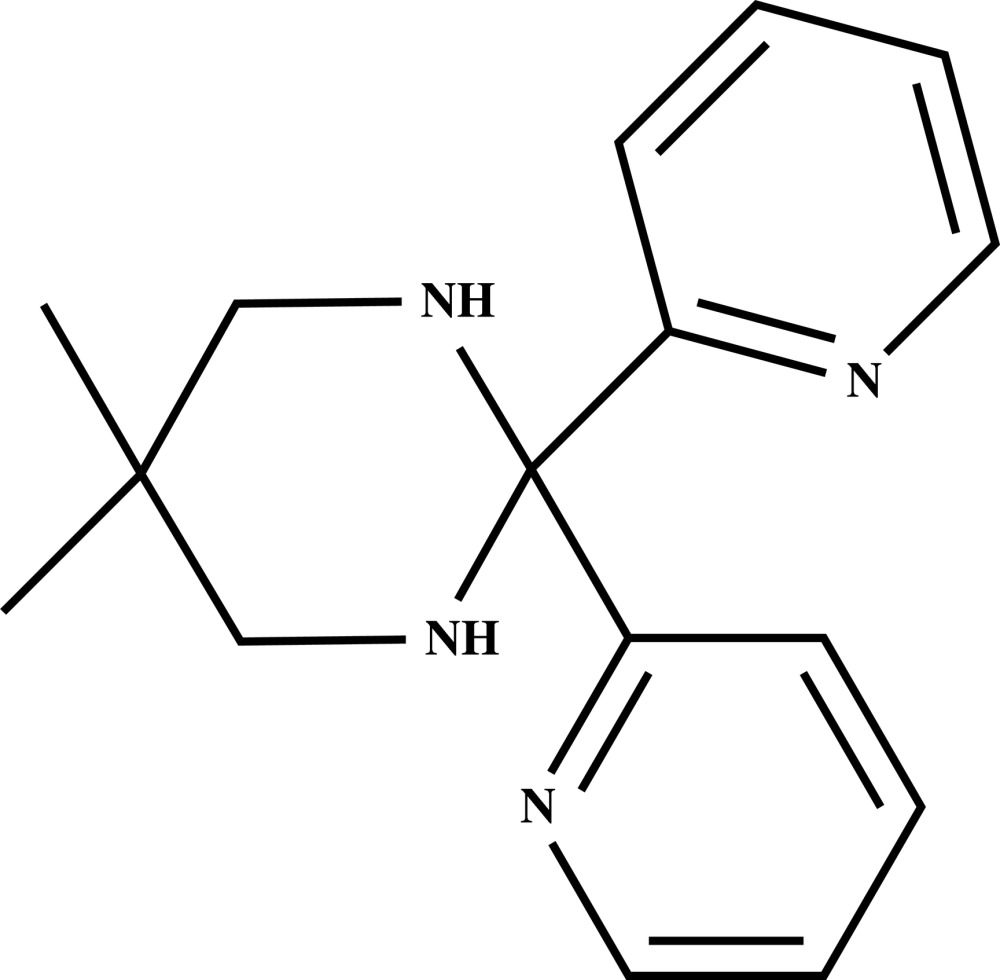


## Experimental

### 

#### Crystal data

C_16_H_20_N_4_
*M* *_r_* = 268.36
Triclinic, 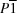

*a* = 8.5535 (7) Å
*b* = 8.7124 (8) Å
*c* = 11.7241 (10) Å
α = 109.824 (2)°
β = 96.444 (2)°
γ = 109.658 (2)°
*V* = 748.33 (11) Å^3^
*Z* = 2
Mo *K*α radiation
μ = 0.07 mm^−1^
*T* = 298 K
0.48 × 0.42 × 0.39 mm

#### Data collection

Bruker SMART APEX CCD area-detector diffractometer
Absorption correction: multi-scan (*SADABS*; Bruker, 2000 ▶) *T*_min_ = 0.966, *T*_max_ = 0.972
8514 measured reflections
2794 independent reflections
2422 reflections with *I* > 2σ(*I*)
*R*_int_ = 0.016

#### Refinement

*R*[*F* ^2^ > 2σ(*F* ^2^)] = 0.040
*wR*(*F* ^2^) = 0.107
*S* = 1.05
2794 reflections
191 parameters
H atoms treated by a mixture of independent and constrained refinement
Δρ_max_ = 0.15 e Å^−3^
Δρ_min_ = −0.17 e Å^−3^

### 

Data collection: *SMART* (Bruker, 2000 ▶); cell refinement: *SAINT* (Bruker, 2000 ▶); data reduction: *SAINT*; program(s) used to solve structure: *SHELXS97* (Sheldrick, 2008 ▶); program(s) used to refine structure: *SHELXL97* (Sheldrick, 2008 ▶); molecular graphics: *SHELXTL* (Sheldrick, 2008 ▶); software used to prepare material for publication: *SHELXTL*, *PARST* (Nardelli, 1995 ▶) and *PLATON* (Spek, 2009 ▶).

## Supplementary Material

Crystal structure: contains datablock(s) global, I. DOI: 10.1107/S1600536812021629/rz2740sup1.cif

Structure factors: contains datablock(s) I. DOI: 10.1107/S1600536812021629/rz2740Isup2.hkl

Supplementary material file. DOI: 10.1107/S1600536812021629/rz2740Isup3.cml

Additional supplementary materials:  crystallographic information; 3D view; checkCIF report

## Figures and Tables

**Table 1:** Hydrogen-bond geometry (Å, °)

*D*—H⋯*A*	*D*—H	H⋯*A*	*D*⋯*A*	*D*—H⋯*A*
N4—H4*A*⋯N2^i^	0.882 (15)	2.469 (15)	3.2048 (14)	141.3 (13)
C4—H4*B*⋯N2	0.93	2.55	3.187 (2)	126

Symmetry code: (i) 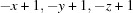.

**Fig. 1.**
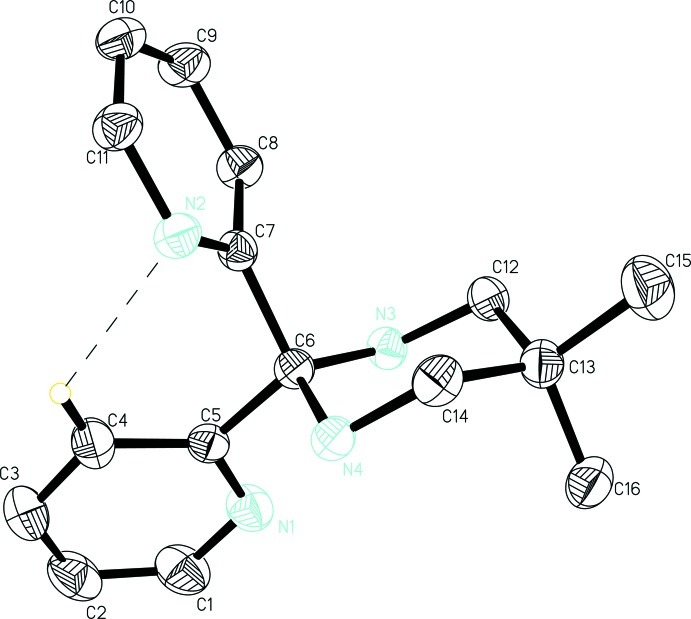
The molecular structure of the title compound with displacement ellipsoids drawn at 30% probability level. Dashed line indicates the intramolecular hydrogen bond. Hydrogen atoms not involved in hydrogen bonds are omitted for clarity.

**Fig. 2.**
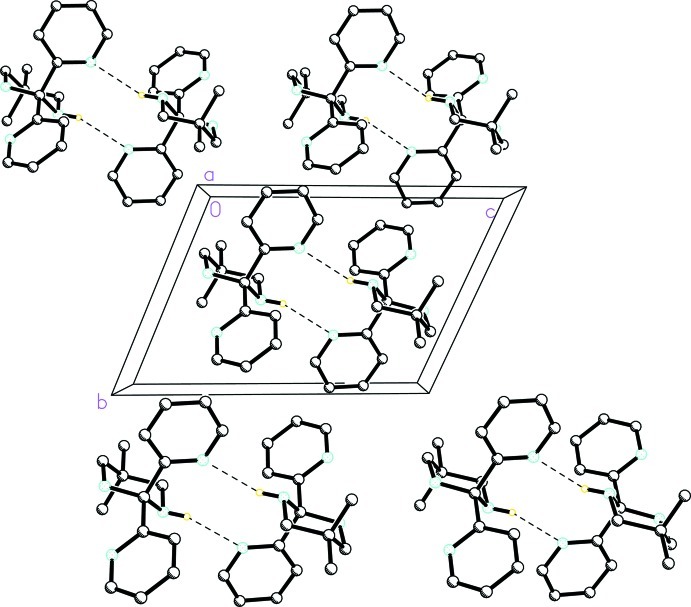
The crystal packing of the title compound viewed along the a axis. Only hydrogen atoms involved in hydrogen bonding (dashed lines) are shown.

## supplementary crystallographic information

### Comment

Condensation of 1,3-diamines with aldehydes and ketones is a well documented reaction for two potential products, classical Schiff bases and hexahydropyrimidine compounds (Al-Resayes, 2009; Song *et al.*, 2010; Jayaratna, & Norman, 2010; Fun & Kia, 2008). Both of these products are extensively utilized as polydentate ligands to synthesize metal coordination complexes. The title compound was obtained during our attempt to synthesize novel ligands in order to study the coordination chemistry of targeted metals.

In the structure of the title compound (Fig. 1), the two pyridine (N1/C1–C5 and N2/C7–C11) rings are *equatorially* and *axially* orientated, respectively, with a dihedral angle of 78.64 (8)° between them. The six membered hexahydropyrimidine ring (N3/N4/C6/C12–C14) adopts a chair conformation, with puckering parameters *Q* = 0.5384 (14) Å, θ = 5.57 (15)° and φ = 179.9 (15)°. All bond lengths (Allen *et al.*, 1987) and angles are in normal range. The molecular conformation is stabilized by a C4—H4B···N2 intramolecular hydrogen bond (Table 1). In the crystal structure, centrosymmetrically related molecules dimerize *via* pairs of N4—H4A···N2 intermolecular hydrogen bonds, generating rings of *R*^2^_2_(10) graph-set motif (Fig. 2).

### Experimental

A solution of 2-dipyridlketone (0.5 g, 2.9 mmol) in anhydrous ethanol (15 mL) was mixed with 2,2-dimethyl-1,3-propanediamine (0.22 mL, 3.0 mmoL) and allowed to reflux for about six hours. The resulting mixture was concentrated under reduced pressure and the title compound was precipitated by the addition of 50 mL of ice cool distilled water. The precipitates were filtered off, washed three times with 20 mL of distilled water, recrystallized in ethanol and allowed to stand at room temperature. After three days, colourless crystals suitable for single-crystal X-ray data collection were obtained (0.63 g, yield 87%). All chemicals were purchased from Acros.

### Refinement

H Atoms on methyl, methylene and methine were positioned geometrically with C—H = 0.96 Å, 0.97 Å and 0.93 Å respectively, and constrained to ride on their parent atoms with *U*_iso_(H) = 1.2*U*_eq_(C) or 1.5*U*_eq_(C) for methyl H atoms. N-bound H atoms were located in a difference Fourier map and refined isotropically (N–H = 0.87 (15)–0.88 (15) Å). A rotating group model was applied to the methyl groups.

### Crystal data

**Table:**

C_16_H_20_N_4_	*Z* = 2
*M**_r_* = 268.36	*F*(000) = 288
Triclinic, *P*1	*D*_x_ = 1.191 Mg m^−^^3^
Hall symbol: -P 1	Mo *K*α radiation, λ = 0.71073 Å
*a* = 8.5535 (7) Å	Cell parameters from 3809 reflections
*b* = 8.7124 (8) Å	θ = 2.6–28.3°
*c* = 11.7241 (10) Å	µ = 0.07 mm^−^^1^
α = 109.824 (2)°	*T* = 298 K
β = 96.444 (2)°	Block, colourless
γ = 109.658 (2)°	0.48 × 0.42 × 0.39 mm
*V* = 748.33 (11) Å^3^	

### Data collection

**Table:**

Bruker SMART APEX CCD area-detector diffractometer	2794 independent reflections
Radiation source: fine-focus sealed tube	2422 reflections with *I* > 2σ(*I*)
Graphite monochromator	*R*_int_ = 0.016
ω scan	θ_max_ = 25.5°, θ_min_ = 1.9°
Absorption correction: multi-scan (*SADABS*; Bruker, 2000)	*h* = −10→10
*T*_min_ = 0.966, *T*_max_ = 0.972	*k* = −10→10
8514 measured reflections	*l* = −14→14

### Refinement

**Table:**

Refinement on *F*^2^	Primary atom site location: structure-invariant direct methods
Least-squares matrix: full	Secondary atom site location: difference Fourier map
*R*[*F*^2^ > 2σ(*F*^2^)] = 0.040	Hydrogen site location: inferred from neighbouring sites
*wR*(*F*^2^) = 0.107	H atoms treated by a mixture of independent and constrained refinement
*S* = 1.05	*w* = 1/[σ^2^(*F*_o_^2^) + (0.0507*P*)^2^ + 0.1284*P*] where *P* = (*F*_o_^2^ + 2*F*_c_^2^)/3
2794 reflections	(Δ/σ)_max_ < 0.001
191 parameters	Δρ_max_ = 0.15 e Å^−^^3^
0 restraints	Δρ_min_ = −0.17 e Å^−^^3^

### Special details

**Table:**

Geometry. All e.s.d.'s (except the e.s.d. in the dihedral angle between two l.s. planes) are estimated using the full covariance matrix. The cell e.s.d.'s are taken into account individually in the estimation of e.s.d.'s in distances, angles and torsion angles; correlations between e.s.d.'s in cell parameters are only used when they are defined by crystal symmetry. An approximate (isotropic) treatment of cell e.s.d.'s is used for estimating e.s.d.'s involving l.s. planes.
Refinement. Refinement of *F*^2^ against ALL reflections. The weighted *R*-factor *wR* and goodness of fit *S* are based on *F*^2^, conventional *R*-factors *R* are based on *F*, with *F* set to zero for negative *F*^2^. The threshold expression of *F*^2^ > σ(*F*^2^) is used only for calculating *R*-factors(gt) *etc*. and is not relevant to the choice of reflections for refinement. *R*-factors based on *F*^2^ are statistically about twice as large as those based on *F*, and *R*- factors based on ALL data will be even larger.

### Fractional atomic coordinates and isotropic or equivalent isotropic displacement parameters (Å^2^)

**Table:**

	*x*	*y*	*z*	*U*_iso_*/*U*_eq_	
N1	0.77584 (15)	0.69943 (16)	0.23880 (11)	0.0588 (3)	
N2	0.52797 (14)	0.27820 (13)	0.38100 (9)	0.0441 (3)	
N3	0.48652 (13)	0.40322 (14)	0.11970 (9)	0.0414 (3)	
N4	0.45050 (13)	0.53799 (13)	0.32684 (9)	0.0408 (3)	
C1	0.9335 (2)	0.8284 (2)	0.28056 (17)	0.0732 (5)	
H1B	0.9634	0.9024	0.2381	0.088*	
C2	1.0538 (2)	0.8587 (2)	0.38177 (17)	0.0741 (5)	
H2B	1.1621	0.9506	0.4075	0.089*	
C3	1.0101 (2)	0.7500 (2)	0.44384 (18)	0.0772 (5)	
H3B	1.0884	0.7667	0.5133	0.093*	
C4	0.84908 (19)	0.6155 (2)	0.40254 (14)	0.0635 (4)	
H4B	0.8173	0.5398	0.4436	0.076*	
C5	0.73434 (16)	0.59355 (16)	0.29919 (11)	0.0418 (3)	
C6	0.54948 (15)	0.45228 (15)	0.25295 (10)	0.0371 (3)	
C7	0.54779 (14)	0.28446 (15)	0.27106 (10)	0.0371 (3)	
C8	0.57432 (17)	0.15174 (17)	0.17968 (12)	0.0464 (3)	
H8A	0.5886	0.1597	0.1041	0.056*	
C9	0.57920 (19)	0.00820 (18)	0.20247 (13)	0.0557 (4)	
H9A	0.5958	−0.0825	0.1421	0.067*	
C10	0.55928 (18)	0.00035 (17)	0.31533 (13)	0.0529 (3)	
H10A	0.5625	−0.0949	0.3332	0.063*	
C11	0.53455 (18)	0.13749 (17)	0.40084 (12)	0.0495 (3)	
H11A	0.5215	0.1325	0.4774	0.059*	
C12	0.30105 (16)	0.30123 (17)	0.07347 (11)	0.0452 (3)	
H12A	0.2747	0.1844	0.0745	0.054*	
H12B	0.2666	0.2844	−0.0131	0.054*	
C13	0.19391 (16)	0.38596 (18)	0.14765 (12)	0.0482 (3)	
C14	0.26589 (16)	0.42847 (18)	0.28538 (12)	0.0481 (3)	
H14A	0.2074	0.4911	0.3369	0.058*	
H14B	0.2444	0.3183	0.2962	0.058*	
C15	0.0071 (2)	0.2539 (2)	0.10458 (17)	0.0744 (5)	
H15A	0.0000	0.1476	0.1154	0.112*	
H15B	−0.0376	0.2248	0.0176	0.112*	
H15C	−0.0588	0.3060	0.1535	0.112*	
C16	0.2069 (2)	0.5537 (2)	0.12841 (15)	0.0634 (4)	
H16A	0.1669	0.5240	0.0407	0.095*	
H16B	0.3243	0.6381	0.1585	0.095*	
H16C	0.1376	0.6048	0.1739	0.095*	
H4A	0.4891 (17)	0.5588 (17)	0.4059 (14)	0.049 (4)*	
H3A	0.5166 (18)	0.502 (2)	0.1080 (13)	0.051 (4)*	

### Atomic displacement parameters (Å^2^)

**Table:**

	*U*^11^	*U*^22^	*U*^33^	*U*^12^	*U*^13^	*U*^23^
N1	0.0570 (7)	0.0547 (7)	0.0576 (7)	0.0107 (6)	0.0113 (6)	0.0271 (6)
N2	0.0578 (6)	0.0430 (6)	0.0357 (5)	0.0239 (5)	0.0133 (5)	0.0164 (4)
N3	0.0465 (6)	0.0455 (6)	0.0314 (5)	0.0176 (5)	0.0093 (4)	0.0156 (4)
N4	0.0509 (6)	0.0441 (6)	0.0308 (5)	0.0250 (5)	0.0112 (4)	0.0130 (4)
C1	0.0675 (10)	0.0612 (9)	0.0758 (11)	0.0059 (8)	0.0196 (9)	0.0294 (8)
C2	0.0477 (8)	0.0607 (10)	0.0834 (12)	0.0063 (7)	0.0121 (8)	0.0102 (9)
C3	0.0560 (10)	0.0700 (10)	0.0780 (11)	0.0142 (8)	−0.0112 (8)	0.0177 (9)
C4	0.0582 (9)	0.0594 (9)	0.0599 (9)	0.0138 (7)	−0.0033 (7)	0.0246 (7)
C5	0.0463 (7)	0.0389 (6)	0.0384 (6)	0.0190 (5)	0.0105 (5)	0.0115 (5)
C6	0.0440 (6)	0.0386 (6)	0.0294 (6)	0.0187 (5)	0.0099 (5)	0.0120 (5)
C7	0.0383 (6)	0.0380 (6)	0.0328 (6)	0.0158 (5)	0.0078 (5)	0.0116 (5)
C8	0.0555 (8)	0.0464 (7)	0.0395 (6)	0.0249 (6)	0.0171 (6)	0.0140 (5)
C9	0.0697 (9)	0.0459 (7)	0.0539 (8)	0.0322 (7)	0.0197 (7)	0.0126 (6)
C10	0.0636 (9)	0.0413 (7)	0.0568 (8)	0.0246 (6)	0.0116 (6)	0.0208 (6)
C11	0.0623 (8)	0.0482 (7)	0.0434 (7)	0.0245 (6)	0.0138 (6)	0.0223 (6)
C12	0.0490 (7)	0.0460 (7)	0.0341 (6)	0.0176 (6)	0.0044 (5)	0.0123 (5)
C13	0.0436 (7)	0.0511 (7)	0.0481 (7)	0.0204 (6)	0.0081 (6)	0.0175 (6)
C14	0.0508 (7)	0.0547 (8)	0.0476 (7)	0.0277 (6)	0.0208 (6)	0.0216 (6)
C15	0.0485 (9)	0.0805 (11)	0.0793 (11)	0.0187 (8)	0.0106 (8)	0.0238 (9)
C16	0.0696 (10)	0.0649 (9)	0.0607 (9)	0.0374 (8)	0.0055 (7)	0.0241 (7)

### Geometric parameters (Å, º)

**Table:**

N1—C5	1.3256 (17)	C8—C9	1.3771 (18)
N1—C1	1.3339 (19)	C8—H8A	0.9300
N2—C7	1.3354 (15)	C9—C10	1.374 (2)
N2—C11	1.3406 (16)	C9—H9A	0.9300
N3—C6	1.4549 (14)	C10—C11	1.3721 (19)
N3—C12	1.4601 (16)	C10—H10A	0.9300
N3—H3A	0.878 (15)	C11—H11A	0.9300
N4—C14	1.4627 (16)	C12—C13	1.5296 (18)
N4—C6	1.4751 (14)	C12—H12A	0.9700
N4—H4A	0.883 (15)	C12—H12B	0.9700
C1—C2	1.367 (3)	C13—C15	1.5230 (19)
C1—H1B	0.9300	C13—C16	1.5231 (19)
C2—C3	1.362 (3)	C13—C14	1.5301 (18)
C2—H2B	0.9300	C14—H14A	0.9700
C3—C4	1.373 (2)	C14—H14B	0.9700
C3—H3B	0.9300	C15—H15A	0.9600
C4—C5	1.3847 (19)	C15—H15B	0.9600
C4—H4B	0.9300	C15—H15C	0.9600
C5—C6	1.5345 (16)	C16—H16A	0.9600
C6—C7	1.5428 (16)	C16—H16B	0.9600
C7—C8	1.3883 (16)	C16—H16C	0.9600
			
C5—N1—C1	117.63 (13)	C8—C9—H9A	120.3
C7—N2—C11	117.43 (11)	C11—C10—C9	117.89 (12)
C6—N3—C12	113.05 (9)	C11—C10—H10A	121.1
C6—N3—H3A	107.0 (9)	C9—C10—H10A	121.1
C12—N3—H3A	110.8 (9)	N2—C11—C10	124.11 (12)
C14—N4—C6	112.88 (9)	N2—C11—H11A	117.9
C14—N4—H4A	109.5 (9)	C10—C11—H11A	117.9
C6—N4—H4A	107.7 (9)	N3—C12—C13	114.99 (10)
N1—C1—C2	124.19 (16)	N3—C12—H12A	108.5
N1—C1—H1B	117.9	C13—C12—H12A	108.5
C2—C1—H1B	117.9	N3—C12—H12B	108.5
C3—C2—C1	117.92 (15)	C13—C12—H12B	108.5
C3—C2—H2B	121.0	H12A—C12—H12B	107.5
C1—C2—H2B	121.0	C15—C13—C16	109.87 (12)
C2—C3—C4	119.16 (16)	C15—C13—C12	109.76 (11)
C2—C3—H3B	120.4	C16—C13—C12	109.97 (11)
C4—C3—H3B	120.4	C15—C13—C14	109.29 (12)
C3—C4—C5	119.43 (15)	C16—C13—C14	111.33 (11)
C3—C4—H4B	120.3	C12—C13—C14	106.57 (10)
C5—C4—H4B	120.3	N4—C14—C13	111.30 (10)
N1—C5—C4	121.67 (13)	N4—C14—H14A	109.4
N1—C5—C6	116.42 (11)	C13—C14—H14A	109.4
C4—C5—C6	121.82 (12)	N4—C14—H14B	109.4
N3—C6—N4	111.25 (9)	C13—C14—H14B	109.4
N3—C6—C5	109.18 (9)	H14A—C14—H14B	108.0
N4—C6—C5	105.31 (9)	C13—C15—H15A	109.5
N3—C6—C7	108.16 (9)	C13—C15—H15B	109.5
N4—C6—C7	113.60 (9)	H15A—C15—H15B	109.5
C5—C6—C7	109.24 (9)	C13—C15—H15C	109.5
N2—C7—C8	122.12 (11)	H15A—C15—H15C	109.5
N2—C7—C6	116.56 (10)	H15B—C15—H15C	109.5
C8—C7—C6	121.24 (10)	C13—C16—H16A	109.5
C9—C8—C7	119.09 (12)	C13—C16—H16B	109.5
C9—C8—H8A	120.5	H16A—C16—H16B	109.5
C7—C8—H8A	120.5	C13—C16—H16C	109.5
C10—C9—C8	119.35 (12)	H16A—C16—H16C	109.5
C10—C9—H9A	120.3	H16B—C16—H16C	109.5
			
C5—N1—C1—C2	−0.5 (3)	C11—N2—C7—C6	−176.83 (10)
N1—C1—C2—C3	0.1 (3)	N3—C6—C7—N2	−151.87 (10)
C1—C2—C3—C4	0.2 (3)	N4—C6—C7—N2	−27.85 (14)
C2—C3—C4—C5	−0.3 (3)	C5—C6—C7—N2	89.39 (12)
C1—N1—C5—C4	0.4 (2)	N3—C6—C7—C8	31.27 (15)
C1—N1—C5—C6	177.15 (12)	N4—C6—C7—C8	155.30 (11)
C3—C4—C5—N1	−0.1 (2)	C5—C6—C7—C8	−87.47 (13)
C3—C4—C5—C6	−176.63 (13)	N2—C7—C8—C9	0.47 (19)
C12—N3—C6—N4	−51.01 (13)	C6—C7—C8—C9	177.15 (11)
C12—N3—C6—C5	−166.81 (9)	C7—C8—C9—C10	−0.6 (2)
C12—N3—C6—C7	74.41 (12)	C8—C9—C10—C11	0.2 (2)
C14—N4—C6—N3	55.48 (13)	C7—N2—C11—C10	−0.4 (2)
C14—N4—C6—C5	173.65 (9)	C9—C10—C11—N2	0.3 (2)
C14—N4—C6—C7	−66.85 (12)	C6—N3—C12—C13	52.20 (14)
N1—C5—C6—N3	29.03 (14)	N3—C12—C13—C15	−170.46 (11)
C4—C5—C6—N3	−154.24 (12)	N3—C12—C13—C16	68.56 (14)
N1—C5—C6—N4	−90.52 (12)	N3—C12—C13—C14	−52.23 (14)
C4—C5—C6—N4	86.21 (14)	C6—N4—C14—C13	−58.94 (13)
N1—C5—C6—C7	147.13 (11)	C15—C13—C14—N4	173.16 (11)
C4—C5—C6—C7	−36.15 (15)	C16—C13—C14—N4	−65.29 (14)
C11—N2—C7—C8	0.00 (18)	C12—C13—C14—N4	54.62 (13)

### Hydrogen-bond geometry (Å, º)

**Table:**

*D*—H···*A*	*D*—H	H···*A*	*D*···*A*	*D*—H···*A*
N4—H4*A*···N2^i^	0.882 (15)	2.469 (15)	3.2048 (14)	141.3 (13)
C4—H4*B*···N2	0.93	2.55	3.187 (2)	126

Symmetry code: (i) −*x*+1, −*y*+1, −*z*+1.

## References

[bb1] Allen, F. H., Kennard, O., Watson, D. G., Brammer, L., Orpen, A. G. & Taylor, R. (1987). *J. Chem. Soc. Perkin Trans. 2*, pp. S1–19.

[bb2] Al-Resayes, S. (2009). *Acta Cryst.* E**65**, o1874. 10.1107/S1600536809025963 PMC2977447 21583568

[bb3] Bruker (2000). *SADABS*, *SMART* and *SAINT* Bruker AXS Inc., Madison, Wisconsin, USA.

[bb4] Fun, H.-K. & Kia, R. (2008). *Acta Cryst.* E**64**, o1840–o1841. 10.1107/S1600536808027232 PMC2960557 21201812

[bb5] Jayaratna, N. B. & Norman, R. E. (2010). *Acta Cryst.* E**66**, o3149. 10.1107/S1600536810045976 PMC3011375 21589448

[bb6] Nardelli, M. (1995). *J. Appl. Cryst.* **28**, 659.

[bb7] Sheldrick, G. M. (2008). *Acta Cryst.* A**64**, 112–122. 10.1107/S0108767307043930 18156677

[bb8] Song, X.-P., Li, G.-C., Wu, C.-Z. & Yang, F.-L. (2010). *Acta Cryst.* E**66**, o1083. 10.1107/S1600536810013127 PMC2979242 21579137

[bb9] Spek, A. L. (2009). *Acta Cryst.* D**65**, 148–155. 10.1107/S090744490804362X PMC2631630 19171970

